# Ototoxic Adverse Drug Reactions: A Disproportionality Analysis Using the Italian Spontaneous Reporting Database

**DOI:** 10.3389/fphar.2019.01161

**Published:** 2019-10-08

**Authors:** Maria Antonietta Barbieri, Giuseppe Cicala, Paola Maria Cutroneo, Eleonora Mocciaro, Laura Sottosanti, Francesco Freni, Francesco Galletti, Vincenzo Arcoraci, Edoardo Spina

**Affiliations:** ^1^Department of Clinical and Experimental Medicine, University of Messina, Messina, Italy; ^2^Sicilian Regional Pharmacovigilance Centre, University Hospital of Messina, Messina, Italy; ^3^Pharmacovigilance Office, Italian Medicines Agency, Rome, Italy; ^4^Department of Adult and Developmental Human Pathology “Gaetano Barresi,” University of Messina, Messina, Italy

**Keywords:** adverse drug reactions, pharmacovigilance, drug-induced ototoxicity, spontaneous reporting, post-marketing data, vestibular disorders, cochlear damage

## Abstract

**Introduction:** The panorama of drug-induced ototoxicity has widened in the last decades; moreover, post-marketing data are necessary to gain a better insight on ototoxic adverse drug reactions (ADRs). The aim of this study was to perform an analysis of ADR reports describing drug-induced ototoxicity from the Italian spontaneous reporting system (SRS).

**Methods:** As a measure of disproportionality, we calculated the reporting odds ratios (RORs) and 95% confidence intervals (CIs) with a case/non-case methodology. Cases were all suspected ADR reports regarding drug-induced ototoxicity collected into the Italian SRS from 2001 to 2017. Non-cases included all other ADRs reported in the same period.

**Results:** Of 325,980 reports, 652 included at least one ototoxic ADR, compared with 325,328 non-cases. Statistically significant adjusted RORs were found for drugs for cardiovascular disorders, urologicals, teriparatide, amikacin, prulifloxacin, rifampicin and isoniazid, cisplatin, hormone antagonists, tacrolimus, pomalidomide, tramadol, and antidepressants. Significant adjusted RORs in relation to tinnitus were also observed for doxazosin (ROR 5.55, 95% CI 2.06–14.93), bisoprolol (4.28, 1.59–11.53), nebivolol (8.06, 3.32–19.56), ramipril (3.96, 2.17–7.23), irbesartan (19.60, 9.19–41.80), betamethasone (4.01, 1.28–12.52), moxifloxacin (4.56, 1.71–12.34), ethambutol (12.25, 3.89–38.57), efavirenz (16.82, 5.34–52.96), sofosbuvir/ledipasvir (5.95, 1.90–18.61), etoposide (7.09, 2.63–19.12), abatacept (6.51, 2.42–17.53), indometacin (6.30, 2.02–19.72), etoricoxib (5.00, 2.23–11.23), tapentadol (4.37, 1.09–17.62), and timolol combinations (23.29, 9.53–56.95). Moreover, significant adjusted RORs for hypoacusis regarded clarithromycin (3.95, 1.86–8.40), azithromycin (10.23, 5.03–20.79), vancomycin (6.72, 2.14–21.11), methotrexate (3.13, 1.00–9.81), pemetrexed (4.38, 1.40–13.76), vincristine (5.93, 1.88–18.70), vinorelbine (21.60, 8.83–52.82), paclitaxel (2.34, 1.03–5.30), rituximab (3.20, 1.19–8.63), interferon alfa-2b (17.44, 8.56–35.53), thalidomide (16.92, 6.92–41.38), and deferasirox (41.06, 20.07–84.01).

**Conclusions:** This study is largely consistent with results from literature. Nevertheless, propafenone, antituberculars, hormone antagonists, teriparatide, tramadol, and pomalidomide are unknown for being ototoxic. Hypoacusis after the use of vinorelbine, methotrexate, and pemetrexed is unexpected, such as tinnitus related with etoposide, nebivolol, betamethasone, abatacept, sofosbuvir/ledipasvir, and tapentadol, but these considerations require further investigation to better define the risk due to the paucity of data. Moreover, physicians should be aware of the clinical significance of ototoxicity and be conscious about the importance of their contribution to spontaneous reporting.

## Introduction

Drug ototoxicity is defined as a temporary or permanent inner ear impairment occurring after a pharmacological treatment that results in hearing and/or balance disorders (Yorgason et al., 2006), depending on the involvement of the cochlear and/or vestibular system, respectively (Ganesan et al., 2018). Cochleotoxicity is characterized by a dysfunction affecting the auditory system that leads to tinnitus or sensorineural damage, while vestibulotoxicity is associated with medical conditions such as dizziness, vertigo, and balance disorders (Lanvers-Kaminsky et al., 2017). These symptoms may appear immediately or progressively and in some cases may be permanent. Clinically, deficits of the cochlear function usually occur much earlier after drug administration than vestibular toxicity (Council for International Organization of Medical Science, 1999).

Ototoxicity can be affected by a high interindividual variability due to differences in terms of age, gender, genetic factors, pharmacokinetics and pharmacodynamics characteristics, comorbidities, and polytherapy. A genetic predisposition can potentially lead to exacerbation of ototoxic effects. Consequently, the identification of genetic variants would be helpful to prevent the development of ototoxicity (Ganesan et al., 2018). Renal impairment constitutes one of the most common conditions that could be associated with audiovestibular disorders because the largest part of ototoxic drugs is eliminated at the kidney level (Meena et al., 2012). Ototoxicity monitoring advanced in clinical practice thanks to the improvement of technologies (Campbell and Le Prell, 2018). Moreover, audiograms, taken before and after the drug treatment, are still the only criterion for establishing a diagnosis of drug-induced hearing loss (Council for International Organization of Medical Science, 1999). In the last two decades, the distortion product otoacoustic emissions (DPOAEs) have established themselves as a highly sensitive method for detecting high-frequency hearing loss and preventing acoustic toxicity, because it could warn about hearing loss before damage of the conversational frequencies (Cevette et al., 2000; Constantinescu et al., 2009). Since most of the ear side effects are not considered as life-threatening conditions, they are undoubtedly underestimated and often overlooked by healthcare professionals (Campbell and Le Prell, 2018). However, they can result in a negative impact on the patient’s quality of life (Brooks and Knight, 2018). The use of ototoxic drugs cannot always be avoided in life-threatening diseases if there are no safer therapeutic alternatives (Campbell and Le Prell, 2018).

The panorama of drug-induced ototoxicity has expanded in the last few decades. The earliest documentation of ototoxicity regards antimalarial drugs, non-steroidal anti-inflammatory drugs (NSAIDs), aminoglycosides, other antimicrobial agents, loop diuretics, and antineoplastic drugs (Schacht and Hawkins, 2006). Several reviews have been issued in the last few years in order to describe drug-related ototoxic effects (Yorgason et al., 2006; Cianfrone et al., 2011; Lanvers-Kaminsky et al., 2017; Ganesan et al., 2018), and almost 600 drugs were identified in an updated guide regarding ototoxicity (Cianfrone et al., 2011). However, the frequency of ear adverse drug reactions (ADRs) and the safety profile of some therapeutic classes remain largely unclear. Pre-marketing clinical trials could assess new drugs that may have the potential of being ototoxic (Campbell and Le Prell, 2018). Nevertheless, several otologic side effects, as well as other ADRs, remain undetected before approval. In this context, post-marketing data are necessary to gain a better insight on drug-induced ototoxicity.

To the best of our knowledge, post-marketing studies designed specifically to identify drug-induced ototoxicity from spontaneous reporting systems (SRSs) are lacking, except a few analyses about specific compounds (Jourde-Chiche et al., 2012; Sagwa et al., 2017). In view of the above findings, the aim of the present study was to conduct an analysis of ADR reports describing drug-induced ototoxicity from the Italian spontaneous ADR reporting database (Italian National Network of Pharmacovigilance, Rete Nazionale di Farmacovigilanza, RNF), by means of a case/non-case methodology.

## Materials and Methods

### Data Source

In Italy, since 2001, reports of suspected ADRs are collected through the RNF, an extensive network managed by the Italian Medicines Agency Agenzia Italiana del Farmaco (AIFA) that links each other, pharmaceutical companies, regional/local health authorities, research centers, and the regional pharmacovigilance centers. This database currently contains more than 420,000 reports.

Each ADR report includes information on the patient (e.g., name/surname initials, age, gender), description of reactions (e.g., time to onset and recovery, seriousness, outcome, dechallenge, rechallenge, relevant laboratory tests), suspected and concomitant drugs (e.g., dosage, frequency and route of administration, therapeutic indication), clinical, history and comorbidities. Drugs are codified using the Anatomical Therapeutic Chemical (ATC) classification (), while suspected ADRs are grouped according to the *Medical Dictionary for Regulatory Activities* (*MedDRA*®) (Brown et al., 1999). Suspected ADRs were defined as serious if they were life-threatening or fatal, required hospitalization (or prolonged existing hospitalization), resulted in persistent or significant disability, or represented a congenital anomaly/birth defect or other medically important conditions (European Medicines Agency, 2017).

### Cases Definition and Selection Criteria

For this analysis, we included all ADR reports regarding drug-induced ototoxicity recorded in the RNF database from December 1, 2001, to December 31, 2017.

All reports having at least one ADR attributed to the clinical definition of ototoxicity according to MedDRA® Preferred Term (PT) were selected and defined as “cases.” Specifically, the following PTs were considered: “acute vestibular syndrome,” “auditory disorder,” “deafness,” “deafness bilateral,” “deafness neurosensory,” “deafness unilateral,” “ear disorder,” “hyperacusis,” “hypoacusis,” “inner ear disorder,” “inner ear inflammation,” “Meniere’s disease,” “motion sickness,” “neurosensory hypoacusis,” “ototoxicity,” “presbyacusis,” “sudden hearing loss,” “tinnitus,” “vertigo,” “vertigo positional,” “vestibular disorder,” and “vestibular neuronitis.” As a reference group (“non-cases”), we selected all other reports in the RNF database which did not contain any ADR related to ototoxicity listed above. Literature cases and duplicate reports were excluded from analysis. Furthermore, since patients with adverse events following immunization (AEFIs) differ largely compared to other drug-related ADRs, reports of vaccine-related adverse events were also excluded. Reports containing “vertigo” as a PT were excluded from this analysis because in spontaneous ADR reports, patients, physicians, and otologists sometimes used the terms vertigo and dizziness interchangeably (Neuhauser, 2016). Although vertigo usually regards vestibular disorders that affect inner ear structures, dizziness can occur with different etiologies including not only vestibular but also cardiovascular, metabolic, psychiatric, or neurologic causes (Drachman and Hart, 1972; Hoffman et al., 1999). In the RNF database, the description of ADRs and corresponding diagnosis were not comprehensive and did not always allow discerning an ototoxic vertigo, leading to some signaling bias against ototoxicity. For this reason, we included in our analysis only reports with a specified vestibular diagnosis (i.e., positional vertigo).

In detail, analysis was performed at the case level, and reports containing more than one event previously mentioned were counted only once.

### Data Analysis

Basal demographic characteristics and drug-related variables of reports were evaluated using descriptive statistical methodology. Absolute and percentage frequencies with 95% confidence intervals (CIs) and medians with interquartile ranges (Q1–Q3) were estimated for categorical and continuous variables, respectively. Since some of the numerical variables resulted as not normally distributed after the application of the Kolmogorov–Smirnov test, a non-parametric approach was used. The Pearson’s chi-squared test for categorical variables and the Mann–Whitney U test for continuous variables were applied to compare characteristics. Only *p* values ≤0.05 were considered as statistically significant.

A case/non-case analysis was performed calculating the crude reporting odds ratio (ROR) and 95% CIs as a measure of signals of disproportionate reporting (SDRs) through a univariate logistic regression model (Egberts et al., 2002). To confirm the previously identified SDRs, we adopted a multivariate logistic regression model (backward procedure ɑ = 5%) based on age, gender, and number of drugs (suspected and concomitant) as predictive factors to calculate the adjusted ROR. The statistical threshold to identify SDRs was defined as the lower bound of the 95% CIs of the ROR >1, with three or more individual reports for each drug–ADR pair. Statistical analysis was conducted using the software “Statistical Package for the Social Science” (SPSS) version 23.0 for Windows (IBM Corp. SPSS Statistics).

Ototoxic ADRs were considered as expected for every drug if acknowledged into the Summary of Product Characteristics (SPCs) available at the time of the study by the European Medicines Agency (EMA) and AIFA websites (Agenzia Italiana del Farmaco Banca Dati Farmaci; European Medicines Agency).

## Results

### Characteristics of Reports

**Figure 1:**
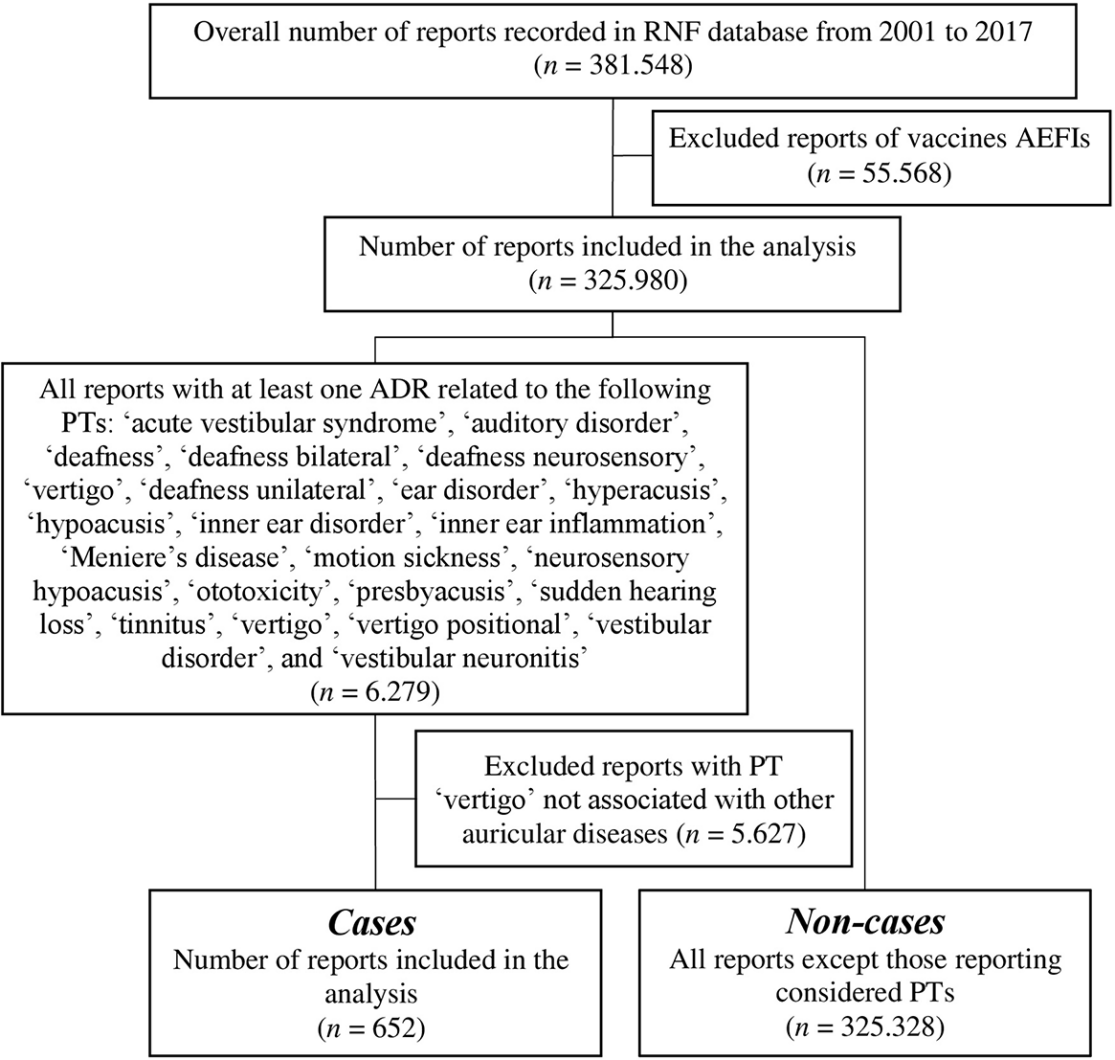
Flowchart of reports selection process.

A total of 381,548 reports of suspected ADRs were collected into the RNF database in the time frame comprehended between December 1, 2001, and December 31, 2017. According to the inclusion and exclusion criteria, the data set regarded 325,980 reports (**Figure 1**). In this analysis, 652 reports (0.2%) included at least one ototoxic ADR, compared with 325,328 non-cases (99.8%).

**Table 1** summarized the main characteristics of selected ADR reports (cases) versus the rest of the RNF database (non-cases) in the same period. Reports were almost equally distributed by sex (female/male ratio 1.1 vs. 1.3); however, a statistically significant difference between cases and non-cases was noticed (*p* = 0.021). In detail, a higher percentage of males was reported in cases compared to non-cases (47.9% vs. 43.3%, respectively). The median [interquartile range Q1–Q3] age was slightly lower for patients with ototoxicity than for patients with other ADRs [60 (45–70) years vs. 61 (44–74)]. Specifically, cases were mostly adults (aged between 18 and 64 years) compared to non-cases (55.1% vs. 48.0%, *p* < 0.001). Overall, 28.7% of ototoxic reports regarded serious ADRs [*n* = 187; in detail, 82 cases (12.6%) concerned medically important conditions, 59 (9.1%) required or prolonged hospitalization, 42 (6.4%) were permanent disabilities, and 4 (0.6%) were life-threatening events]. Nevertheless, cases showed a lower seriousness when compared to the reference group (28.7% vs. 34.8%, *p* = 0.008). Focusing on ADR outcome, cases had a higher statistically significant proportion of ADRs “not yet recovered” or “recovered with sequelae” (for both *p* < 0.001) than non-cases. The median number of suspected/concomitant drugs did not differ between groups (*p* = 0.293) (**Table 1**).

**Table 1:** Description of ototoxic adverse drug reaction (ADR) reports in the Italian spontaneous reporting system database during the period 2001–2017.

Characteristic	Cases (*n* = 652)	Non-cases (*n* = 325,328)	*p* value^a^
Sex			
Females	337 (51.7)	182.226 (56.0)	0.021
Males	312 (47.9)	140.753 (43.3)	
Missing value	3 (0.5)	2.349 (0.7)	
Female/male ratio	1.1	1.3	
Median age *n* (IQR: Q1–Q3)	60 (45–70)	61 (44–74)	0.088
Age categories (years)			
<18	17 (2.6)	19.318 (5.9)	<0.001
18–64	359 (55.1)	156.307 (48.0)	
≥65	245 (37.6)	139.456 (42.9)	
Missing value	31 (4.8)	10.247 (3.2)	
Serious ADRs			
Serious	187 (28.7)	113.127 (34.8)	0.008
Not serious	408 (62.6)	195.525 (60.1)	
Not available	57 (8.7)	16.676 (5.1)	
Outcome of ADRs			
Complete recovery	203 (31.1)	136.366 (41.9)	<0.001
Improvement	131 (20.1)	92.945 (28.6)	<0.001
Not yet recovered	128 (19.6)	20.121 (6.2)	<0.001
Recovered with sequelae	23 (3.5)	5.400 (1.7)	<0.001
Death	0 (0.0)	3.756 (1.1)	–
Missing value	167 (25.6)	66.740 (20.5)	
Median number of drugs *n* (IQR: Q1–Q3)	2 (1–3)	2 (1–4)	0.293

ADR, adverse drug reaction; IQR, interquartile range; Q1, first quartile; Q3, third quartile.

a Patients with ototoxic ADRs versus patients with other ADRs (Pearson’s chi-squared test or Mann–Whitney U test).

In general, drug classes most frequently involved in cases of ototoxic ADRs were other antineoplastic agents (*n* = 72), immunosuppressants (*n* = 41), quinolone antibacterials (*n* = 32), and antidepressants (*n* = 30). More than 50% of reports involved tinnitus (*n* = 358; 54.9%). Other relevant ototoxic ADRs were hypoacusis, including neurosensory hypoacusis (*n* = 213; 32.7%) and deafness, including deafness bilateral, deafness neurosensory, deafness unilateral, and sudden hearing loss (*n* = 21; 3.2%). In particular, drug classes most frequently reported for tinnitus, hypoacusis, or deafness induction were other antineoplastic agents (**Supplementary Table 1**).

### Disproportionality Analyses

The ototoxic profile of drug classes, examined using ROR as a disproportionality measure, is displayed in **Supplementary Table 2**. The adjusted ROR values showed a statistical significance in relation to the following drugs: quinolones, macrolides and aminoglycosides, antidepressants, plant alkaloids and other natural products, beta-blockers, plain angiotensin-converting enzyme (ACE) inhibitors, agents acting on the renin–angiotensin system (RAS), selective Ca^2+^ channel blockers and peripherally acting antiadrenergic agents, urologicals, hormone antagonists, parathyroid hormones and anterior pituitary lobe hormones, antiglaucoma preparations and miotics, antituberculars and antimalarials, other otologicals, antimigraine preparations, and all other therapeutic products (e.g., iron-chelating agents). Tinnitus and hypoacusis were the most reported ototoxic adverse effects for quinolones (*n* = 18, *n* = 9, respectively), macrolides (*n* = 7,* n* = 15, respectively), aminoglycosides (*n* = 8, *n* = 12, respectively), plant alkaloids (*n* = 12,* n* = 16, respectively), urologicals (*n* = 4, *n* = 3, respectively), and antituberculars (*n* = 3, *n* = 2, respectively, one of which related to a multidrug-resistance tuberculosis). Few cases of hearing loss were related to aminoglycosides (*n* = 3). Antidepressants were frequently associated with tinnitus (*n* = 20), hypoacusis, and positional vertigo (*n* = 5 in both cases). Beta-blockers, plain ACE inhibitors, RAS-acting agents, selective Ca^2+^ channel blockers, peripherally acting antiadrenergic agents, hormone antagonists, as well as antiglaucoma preparations and miotics mostly regarding timolol in combinations were mainly involved with the occurrence of tinnitus (*n* = 15, *n* = 14, *n* = 10, *n* = 9, *n* = 4,* n* = 7, and *n* = 7, respectively). Parathyroid hormone–related cases regarded only teriparatide and included tinnitus (*n* = 2), positional vertigo (*n* = 2), or hearing disorders (*n* = 3, including hypoacusis and deafness), while anterior pituitary lobe hormones were mostly involved with the onset of hypoacusis (*n* = 3). Other therapeutic products mainly concerned deferasirox-related cases of hypoacusis (*n* = 10). Few numbers of ototoxic ADRs were associated with antimalarials, other otologicals, and antimigraine preparations.

The adjusted RORs for single active substances regarding ototoxicity and relevant PTs with significance are shown in **Table 2** (the full crude and adjusted ROR data for active substances and relevant PTs tinnitus and hypoacusis are available in **Supplementary Tables 3**, **4**, and **5**). Concerning quinolones, a significant adjusted ROR was observed for prulifloxacin (ROR 8.64, 95% CI 3.21–23.30) related to ototoxicity and for moxifloxacin regarding tinnitus (4.56, 1.71–12.34). Clarithromycin (3.95, 1.86–8.40) and azithromycin (10.23, 5.03–20.79) regarding macrolides showed a statistically significant disproportionality for the onset of hypoacusis, as well as amikacin (128.65, 66.37–249.37) among aminoglycosides, that had a significant adjusted ROR also related to tinnitus (39.52, 16.02–97.52). Paroxetine, sertraline, duloxetine, escitalopram, and vortioxetine were antidepressants with significant ototoxic adjusted RORs (2.67, 1.10–6.45; 2.99, 1.24–7.24; 3.11, 1.16–8.33; 3.15, 1.17–8.44; and 13.18, 4.18–41.58, respectively). The adjusted RORs remained significant for the onset of tinnitus only with paroxetine and sertraline. As regards plant alkaloids and podophyllotoxin derivatives, the adjusted RORs related to hypoacusis were significant for vincristine (5.93, 1.88–18.70), vinorelbine (21.60, 8.83–52.82), and paclitaxel (2.34, 1.03–5.30), while etoposide had a significant ROR for the onset of tinnitus (7.09, 2.63–19.12). In our cases, all antineoplastic drugs listed above had never been reported alone but always in association with other agents, such as platinum compounds (*n* = 11), anthracyclines (*n* = 4), or rituximab (*n* = 2). Focusing on drugs acting on the cardiovascular system, ototoxic significant RORs emerged for bisoprolol (2.84, 1.17–6.87), nebivolol (4.40, 1.82–10.66), metoprolol (4.97, 1.59–15.54), ramipril (2.50, 1.44–4.33), irbesartan (10.44, 4.91–22.17), and doxazosin (3.73 1.54–9.03). All of these drugs listed above except for metoprolol had significant adjusted RORs for the onset of tinnitus. As for urologicals, we observed significant RORs for sildenafil and tadalafil (5.96, 1.47–24.18 and 9.36, 3.85–22.76, respectively). Looking at hormone antagonists, tamoxifen and anastrozole showed a significant adjusted ROR (6.33, 2.02–19.85 and 3.45, 1.10–10.79, respectively). Concerning parathyroid hormones, we observed a possible association with ototoxicity for teriparatide (2.25, 1.06–4.76). Iron-chelating agents showed a positive adjusted ROR for deferasirox in relation with hypoacusis (41.06, 20.07–84.01). Antiglaucoma preparations and miotics, in particular, timolol in combination, had a statistically significant disproportion related to tinnitus (23.29, 9.53–56.95). Possible signals of disproportionality were identified for antitubercular agents, including ethambutol (and the rifampicin and isoniazid combination). Moreover, ethambutol was the only antitubercular with a significant association with tinnitus (12.25, 3.89–38.57). Antituberculars were not administered alone but always in association with other drugs (e.g., amikacin, moxifloxacin, or azithromycin).

**Table 2:** Adjusted reporting odds ratio (ROR) for the association of active substances with ototoxicity and significant respective Preferred Terms (PTs).

Drug Classes	Active Substances	Ototoxic ADR reports (*n*)^a^	Other ADR reports (*n*)	Adjusted ROR^b^ (95% CI)	Relevant PT (*n*)^a^ with significant adjusted ROR (95% CI)	Unexpected Ototoxic ADR^c^
Drugs for peptic ulcer and gastro-esophageal reflux disease	Omeprazole	3	685	2.30 (0.74–7.17)		
	Lansoprazole	3	1.299	1.21 (0.39–3.76)		
Antithrombotic agents	Warfarin	5	13.630	0.18 (0.07–0.43)		
	Acetylsalicylic acid	9	9.179	0.48 (0.25–0.93)		
	Clopidogrel	9	4.199	1.15 (0.60–2.23)		
Antiarrhythmics, classes I and III	Propafenone	3	284	***5.56 (1.78–17.40)***		
Antiadrenergic agents, peripherally acting	Doxazosin	5	708	***3.73 (1.54–9.03)***	Tinnitus (*n* = 4; 5.55, 2.06–14.93)	No
High-ceiling diuretics	Furosemide	3	1.746	0.94 (0.30–2.94)		
Diuretics and potassium-sparing agents in combination	Hydrochlorothiazide and potassium-sparing agents	3	433	***3.72 (1.19–11.62)***		
Beta-blocking agents	Metoprolol	3	322	***4.97 (1.59–15.54)***		
	Bisoprolol	6	981	***2.84 (1.17–6.87)***	Tinnitus (*n* = 5; 4.28, 1.59–11.53)	No
	Nebivolol	5	595	***4.40 (1.82–10.66)***	Tinnitus (*n* = 5; 8.06, 3.32–19.56)	Yes
Selective calcium channel blockers with mainly vascular effects	Amlodipine	3	1.437	1.10 (0.35–3.41)		
ACE inhibitors, plain	Ramipril	13	2.778	***2.50 (1.44–4.33)***	Tinnitus (*n* = 11; 3.96, 2.17–7.23)	No
Agents acting on the renin–angiotensin system	Irbesartan	7	362	10.44 (4.91–22.17)	Tinnitus (*n* = 7; 19.60, 9.19–41.80)	No
Angiotensin II antagonists, combinations	Losartan and diuretics	3	231	***6.83 (2.18–21.40)***		
Lipid-modifying agents, plain	Atorvastatin	8	2.715	1.58 (0.79–3.19)		
Urologicals	Sildenafil	3	172	***5.96 (1.47–24.14)***		
	Tadalafil	5	309	***9.36 (3.85–22.76)***		
Corticosteroids for systemic use, plain	Betamethasone	3	714	2.20 (0.71–6.85)	Tinnitus (*n* = 3; 4.01, 1.28–12.52)	Yes
Parathyroid hormones and analogues	Teriparatide	7	1.899	***2.25 (1.06–4.76)***		
Beta-lactam antibacterials, penicillins	Amoxicillin	3	6.425	0.23 (0.07–0.71)		
	Amoxicillin and beta-lactamase inhibitor	6	13.236	0.22 (0.10–0.49)		
Macrolides, lincosamides, and streptogramins	Clarithromycin	13	2.874	***2.34 (1.35–4.06)***	Hypoacusis (*n* = 7; 3.95, 1.86–8.40)	No
	Azithromycin	11	1.297	***4.47 (2.45–8.13)***	Hypoacusis (*n* = 8; 10.23, 5.03–20.79)	No
Aminoglycoside antibacterials	Amikacin	16	119	75.05 (44.07–127.79)	Hypoacusis (*n* = 10; 128.65, 66.37–249.37) Tinnitus (*n* = 5; 39.52, 16.02–97.52)	No
Quinolone antibacterials	Ciprofloxacin	10	3.020	1.37 (0.68–2.75)		
	Levofloxacin	12	5.184	1.20 (0.68–2.12)		
	Moxifloxacin	4	816	2.53 (0.94–6.77)	Tinnitus (*n* = 4; 4.56, 1.71 –12.34)	No
	Prulifloxacin	4	237	***8.64 (3.21–23.30)***		
Other antibacterials	Vancomycin	4	671	***3.13 (1.17–8.40)***	Hypoacusis (*n* = 3; 6.72, 2.14–21.11)	No
Drugs for treatment of tuberculosis	Ethambutol	4	252	***8.60 (3.18–23.22)***	Tinnitus (*n* = 3; 12.25, 3.89–38.57)	Yes
	Rifampicin and isoniazid	3	142	***10.97 (3.48–34.52)***		
Direct-acting antivirals	Efavirenz	3	171	***8.86 (2.82 –7.85)***	Tinnitus (*n* = 3; 16.82, 5.34–52.96)	No
	Ribavirin	6	3.379	0.92 (0.41–2.05)		
	Sofosbuvir and ledipasvir	3	512	**3.19 (1.02–9.95)**	Tinnitus (*n* = 3; 5.95, 1.90–18.61)	Yes
Antimetabolites	Methotrexate	3	1.507	1.09 (0.35–3.41)	Hypoacusis (*n* = 3; 3.13, 1.00–9.81)	Yes
	Pemetrexed	5	1.101	2.32 (0.96–5.60)	Hypoacusis (*n* = 3; 4.38, 1.40–13.76)	Yes
	Fluorouracil	5	3.362	0.73 (0.30–1.75)		
Plant alkaloids and other natural products	Vincristine	4	713	***3.08 (1.15–8.29)***	Hypoacusis (*n* = 3; 5.93, 1.88–18.70)	No
	Vinorelbine	5	390	***6.75 (2.78–16.36)***	Hypoacusis (*n* = 5; 21.60, 8.83–52.82)	Yes
	Etoposide	4	569	***3.71 (1.38–9.96)***	Tinnitus (*n* = 4; 7.09, 2.63–19.12)	Yes
	Paclitaxel	10	4.521	1.20 (0.64–2.25)	Hypoacusis (*n* = 6; 2.34, 1.03–5.30)	No
	Docetaxel	4	2.488	0.84 (0.31–2.24)		
Other antineoplastic agents	Cisplatin	34	1.731	***10.16 (7.09–14.56)***	Hypoacusis (*n* = 19; 19.29, 11.99–31.03) Tinnitus (*n* = 13; 6.18, 3.38–11.30)	No
	Rituximab	5	2.036	1.30 (0.54–3.14)	Hypoacusis (*n* = 4; 3.20, 1.19–8.63)	No
	Carboplatin	5	2.488	1.08 (0.45–2.60)		
	Oxaliplatin	6	5.281	0.58 (0.26–1.30)		
	Bortezomib	3	910	1.71 (0.55–5.34)		
Hormone antagonists and related agents	Tamoxifen	3	265	***6.33 (2.02–19.85)***		
	Anastrozole	4	476	***3.45 (1.10–10.79)***		
Immunostimulants	Interferon alfa-2b	9	724	***6.43 (3.31–12.47)***	Hypoacusis (*n* = 8; 17.44, 8.56–35.53)	No
	Glatiramer acetate	5	1.258	1.46 (0.47–4.56)		
Immunosuppressants	Abatacept	4	823	***3.67 (1.37–9.87)***	Tinnitus (*n* = 4; 6.51, 2.42–17.53)	Yes
	Infliximab	4	2.239	0.92 (0.35–2.48)		
	Adalimumab	4	1.669	1.36 (0.51–3.64)		
	Tacrolimus	3	152	***10.55 (3.35–33.22)***		
	Thalidomide	7	535	***7.44 (3.51–15.76)***	Hypoacusis (*n* = 5; 16.92, 6.92–41.38)	No
	Lenalidomide	3	3.444	0.45 (0.15–1.40)		
	Pomalidomide	3	173	***10.64 (3.38–33.48)***		
Anti-inflammatory and antirheumatic products, non-steroids	Indometacin	4	449	***4.71 (1.76–12.65)***	Tinnitus (*n* = 3; 6.30, 2.02–19.72)	No
	Diclofenac	4	3.100	0.49 (0.16–1.54)		
	Ketoprofen	3	5.077	0.29 (0.09–0.90)		
	Etoricoxib	6	1.130	***2.77 (1.24–6.21)***	Tinnitus (*n* = 6; 5.00, 2.23–11.23)	No
Antigout preparations	Allopurinol	3	1.826	0.58 (0.15–2.34)		
Opioids	Tramadol	8	2.031	***2.07 (1.03–4.17)***		
	Tapentadol	3	461	2.45 (0.61–9.83)	Tinnitus (*n* = 3; 4.37, 1.09–17.62)	Yes
Other analgesics and antipyretics	Acetylsalicylic acid	5	1.523	1.64 (0.68–3.96)		
Antiepileptics	Carbamazepine	3	1.075	0.98 (0.24–3.92)		
	Oxcarbazepine	3	388	2.75 (0.68–11.06)		
	Valproic acid	5	1.205	1.75 (0.65–4.69)		
	Levetiracetam	3	538	3.03 (0.97–9.46)		
	Pregabalin	3	1.037	1.05 (0.26–4.22)		
Antipsychotics	Quetiapine	3	1.766	0.91 (0.29–2.82)		
Antidepressants	Paroxetine	6	996	***2.67 (1.10–6.45)***	Tinnitus (*n* = 6; 4.79, 1.98–11.61)	No
	Duloxetine	5	722	***3.11 (1.16–8.33)***		
	Escitalopram	4	699	***3.15 (1.17–8.44)***		
	Sertraline	5	893	***2.99 (1.24–7.24)***	Tinnitus (*n* = 4; 4.34, 1.61–11.65)	No
	Vortioxetine	3	141	***13.18 (4.18–41.58)***		
Antiglaucoma preparations and miotics	Timolol, combinations	5	216	***12.70 (5.21–30.95*** *)*	Tinnitus (*n* = 5; 23.29, 9.53–56.95)	No
All other therapeutic products	Deferasirox	10	328	***14.66 (7.52–28.58)***	Hypoacusis (*n* = 9; 41.06, 20.07–84.01)	No
X-ray contrast media, iodinated	Iomeprol	3	3.714	0.40 (0.13–1.23)		

ATC, Anatomical Therapeutic Chemical classification system; CI, confidence interval; ACE, angiotensin-converting enzyme.

a Only ADR reports for three or more were considered.

b Adjusted for age, sex, and number of drugs.

c Unexpected ADRs based on the definition of “unexpected adverse reaction.”

Bold-italic indicate the statistically significant adjusted ROR values.

Other SDRs of general ototoxicity regarded: propafenone, hydrochlorothiazide and potassium-sparing agents, losartan and diuretics, cisplatin, tacrolimus, pomalidomide, and tramadol. A significant association with hypoacusis was observed for: vancomycin, methotrexate, pemetrexed, rituximab, interferon alfa-2b, and thalidomide. Focusing on tinnitus, betamethasone, efavirenz, sofosbuvir/ledipasvir, abatacept, indometacin, etoricoxib, and tapentadol were drugs presenting a significant disproportionality (**Table 2**).

## Discussion

This is the first study comparing the ototoxic profile of drugs based on the Italian SRS database. To the best of our knowledge, only recently a few studies from SRSs are becoming available for single drug classes (Jourde-Chiche et al., 2012; Sagwa et al., 2017).

We noticed a statistically significant difference in terms of gender and age between cases and non-cases. Focusing on gender, a higher percentage of males was reported in cases compared to non-cases. From several published studies, a higher prevalence of tinnitus or hearing loss in males compared to females was observed (Agrawal et al., 2008; Wu et al., 2015). Concerning patients’ age, we found that the adult group was mostly affected by ototoxicity compared to the rest of the SRS database. This could probably find an explanation in the main use of ototoxic drugs, such as antineoplastics or antibiotics, in adult patients. However, literature data reported a greater sensitivity to ototoxicity for both older and pediatric patients (Aguilar-Markulis et al., 1981; Brummett, 1993; Sagwa et al., 2017; Sogebi et al., 2017). For the elderly patients, this could find an explanation in the apoptotic loss of the auditory sensory hair cells of the organ of Corti (Sagwa et al., 2017). The percentage of serious ADRs was lower for ototoxic reports than non-cases. This was probably due to the onset of mild ear ADRs that are not considered as life-threatening conditions (Campbell and Le Prell, 2018). Drug-induced auricular disorders, such as tinnitus, hypoacusis, and hearing loss, may range from temporary to permanent conditions (Ganesan et al., 2018). Concerning ototoxic ADRs’ outcome, most patients recovered completely, but a significant proportion of them “recovered with sequelae” or were not recovered at the time of reporting. Median number of drugs was similar between cases and non-cases.

The present analysis supports the hypothesis that some drugs are potentially ototoxic and more frequently associated with ear disorders than others. Generally, our findings are consistent with information reported in SPCs and literature (Agenzia Italiana del Farmaco Banca Dati Farmaci; European Medicines Agency). Quinolones and macrolides are among the ototoxicity-related drugs (Cervin and Wallwork, 2014; Cianfrone et al., 2011). Ototoxicity is also one of the most common forms of toxicity encountered with aminoglycosides (Bitner-Glindzicz and Rahman, 2007; Ganesan et al., 2018). Some studies estimated the overall incidence of aminoglycoside ototoxicity in patients at 7.5%. In detail, the incidence of cochlear and vestibular toxicity for gentamicin are 8% and 14%, respectively, whereas for amikacin, they are 5% and 13%, respectively (Govaerts et al., 1990). In our research, urologicals, in particular, drugs used in erectile dysfunction, tadalafil and sildenafil, were significantly associated with ototoxicity, which is acknowledged in the literature (Barreto and Bahmad, 2013). Ototoxicity results were statistically significant for antimalarials too. A French pharmacovigilance study stated that audiovestibular ADRs were 2.6% of all antimalarial drug-related spontaneous ADR reports (Jourde-Chiche et al., 2012). Antidepressants are notorious for causing tinnitus and positional vertigo (Clewes, 2012). Within other therapeutic products, we observed a significant proportion of cases concerning deferasirox-induced hypoacusis. It is well known that iron-chelating agents have been associated with a predominantly high frequency of sensorineural hearing loss (Osma et al., 2015). As for timolol used in combination for antiglaucoma therapy, the onset of tinnitus is known and could be related to the systemic beta-blockade (Frishman et al., 1994). Anterior pituitary lobe hormones, in particular somatropin agonists, are also known to be ototoxic. Hearing loss is common in children with growth hormone deficiency, with a predisposition to be bilateral (Muus et al., 2017). Antimigraine preparations were related with the onset of ototoxic ADRs (Cianfrone et al., 2011). Moreover, in accordance with corresponding SPCs (Agenzia Italiana del Farmaco Banca Dati Farmaci; European Medicines Agency), we found a positive association for vancomycin, efavirenz, cisplatin, rituximab, interferon alfa-2b, thalidomide, tacrolimus, indometacin, and etoricoxib.

Conversely to the abovementioned, we noticed several differences worthy of discussion. Plant alkaloids and podophyllotoxin derivatives were significantly associated with the onset of ototoxic ADRs. Although deafness is reported into vincristine and paclitaxel labels as an ear disorder, hypoacusis and tinnitus for vinorelbine and etoposide, respectively, are unexpected. Nevertheless, vinca alkaloids and etoposide are frequently used along with cisplatin, a known ototoxic drug, thus making it difficult to separate the relative effects of the combination agents. In a previous trial, ototoxicity was identified with an incidence from 1% with single-agent vinorelbine to 10% with vinorelbine plus cisplatin (Le Chevalier et al., 1994). Moreover, in US post-marketing data about vinorelbine, hearing impairment has been reported (Food and Drug Administration, 2014). As far as etoposide was concerned, in a randomized trial, tinnitus and/or hearing loss was present in 24.5% of patients treated with etoposide and cisplatin versus 2.8% of patients in treatment with single-agent etoposide (Rosso et al., 1990). In the disproportionality analysis, we found a statistically significant ROR for drugs used in cardiovascular disorders (beta-blockers, plain ACE inhibitors, RAS-acting agents, selective Ca^2+^ channel blockers, and alpha-blockers) but also for the single active substances (propafenone, doxazosin, metoprolol, bisoprolol, nebivolol, ramipril, irbesartan, losartan and diuretics, and hydrochlorothiazide and potassium-sparing agents). In considering ototoxicity, a previous review reported ACE inhibitors, beta-blockers, diuretics, RAS agents, and Ca^2+^ channel blockers as possible ototoxic medications (Cianfrone et al., 2011). Activation of the sense of hearing is an important component of the fight-or-flight reaction (Fauser et al., 2004). β1 adrenoceptors are present in inner ear epithelial cells, in the cochlea, and in the vestibular system, specifically in the strial marginal cells of the strial vascularis (Wangemann et al., 2000). Decreasing the function of β receptors may result in defects in hearing (Al-Ghamdi et al., 2018). Our results showed a higher association for antihypertensive drugs with the onset of tinnitus. The onset of tinnitus could result from a decrease in cochlear blood flow when blood pressure decreases and vasomotor autoregulation is impaired. This process might be drastically increased by the concomitant use of vasodilators, which could further reduce the autoregulation of cochlear microcirculation. On the other hand, arterial hypertension also could be considered as a possible cause of tinnitus (Johnson and Zonderman, 1948). Treatment with drugs that increase peripheral vascular tone (beta-blockers) or activate the RAS might contribute to the development of tinnitus in the hypertensive population (Borghi et al., 2005). We observed that tinnitus, related to doxazosin, hydrochlorothiazide and potassium-sparing agents, metoprolol, ramipril, irbesartan, losartan, and diuretics, is included in their labels. However, the onset of tinnitus is not reported for nebivolol. We noticed two cases of tinnitus and one case of hypoacusis after propafenone use, but the low number of reports prevents confirmation of its potential ototoxicity. Several ototoxic reports were retrieved for hormone antagonists, resulting in a statistically significant ROR for anastrozole and tamoxifen. No mention about occurrence of ear disorders is available for these drugs. Studies about estrogen’s protective effects on auditory function based on the cochlear localization of estrogen receptors are available (Hultcrantz et al., 2006). This could explain the ototoxic profile of anti-estrogens, but data are still too weak to be confirmed. Teriparatide, notorious for causing positional vertigo, was also related to tinnitus and hearing loss. Moreover, previous studies demonstrated a probable association between osteoporosis and hearing loss (Upala et al., 2017), as well as a remarkably higher incidence of tinnitus in patients with osteoporosis (Kahveci et al., 2014). Few cases regarded other otologicals, and the onset of ototoxicity could be due to the route of administration or to a worsening of a pre-existing condition. Statistical analysis highlighted a positive association with ototoxicity for antitubercular drugs, and in particular for the correlation of ethambutol with tinnitus, but the totality of detected ADRs is not yet acknowledged. In an observational study, hospitalized patients in treatment with antitubercular agents experienced mostly ototoxicity (Gülbay et al., 2006). Nevertheless, in multidrug-resistant tuberculosis, where ototoxicity was reported as the second most common ADR, there is a possibility of enhanced effects of interaction with other concomitant and potentially ototoxic drugs that were used in the regimen, as we found in our results (Prasad et al., 2016).

No information about betamethasone-induced tinnitus is available. However, in 14 cases of tinnitus were related to betamethasone, and only 3 cases belonged to our SRS database (). Three cases of tinnitus were retrieved for sofosbuvir/ledipasvir. To the best of our knowledge, no information about ototoxicity associated with direct-acting antivirals against hepatitis C is available in the literature. Actually, in VigiBase, 97 reports of tinnitus were related to sofosbuvir/ledipasvir (). Significant methotrexate-related onset of hypoacusis in patients with inflammatory arthritis is unexpected according to SPCs. A possible explanation could be the presence of an extra-articular manifestation in rheumatoid arthritis, known as rheumatoid nodules, from which the ears could be affected. The administration of methotrexate could cause rheumatoid nodules and increase their development (Tilstra and Lienesch, 2015). Moreover, a dose relation between hearing loss and using methotrexate has been observed (Dikici et al., 2009). Pemetrexed had a significant correlation with hypoacusis. In our cases, it was always reported in association with the already known ototoxic cisplatin. Nevertheless, in a pemetrexed risk management plan, hypoacusis is considered as an important potential risk because it is unclear what role pemetrexed may have played in this event (European Medicines Agency, 2015). No information about abatacept-induced tinnitus is available. However, the parent company included tinnitus into the less common clinical trial ADRs (<1.0%). Possible onset of hypoacusis is not acknowledged for pomalidomide. Actually, 50 cases of pomalidomide-induced hypoacusis were retrieved from and only 2 cases belonged to our SRS database (). Tramadol is notorious for causing positional vertigo. Tinnitus is known only as a withdrawal symptom, even if it was infrequently reported in post-marketing experience with an unknown causal relationship (Food and Drug Administration, 2003). In our cases, tinnitus occurred only during pharmacological treatment. Tinnitus with tapentadol is unexpected. Actually, in VigiBase, only 13 reports of tapentadol-induced tinnitus are available ().

### Limits and Strengths

The SRS is the most common method used in pharmacovigilance and the best one to generate signal on new or rare ADRs (Galatti et al., 2005). Our study has some strengths and limitations. The main strength is that we conducted the first overview of all drug-induced ototoxicity from an SRS database. ROR computing does not allow a quantification of the true risk of ototoxicity, and it is often calculated on a limited number of cases; it only suggests a statistically significant disproportionality of specific drug–ADR pairs, which should be further investigated for signal validation (Raschi et al., 2019). We believe that our findings reflect real differences in the relative ototoxicity of these drugs in the real world compared to pre-marketing authorization studies. When feasible, we evaluated causality of each single report, literature data, and biological plausibility to substantiate potential signals with significant RORs. Despite this, in a few cases, the uncertainty remains, and we cannot rule out the presence of other possible confounding variables that might have contributed to the occurrence of ototoxicity, such as the role of comorbidities and co-medications, because of limited clinical data. Moreover, the uncertainty of the potential causal relationship between drug and ADR, as well as the difficulty to completely understand the biological plausibility and to characterize the real onset of cochlear and vestibular toxicity because of the different drug mechanisms of action or the missing data about therapy dates or about audiometric exams for cochlear function, often represents major limitations. Furthermore, the existence of under- or over-reporting of suspected ADRs and missing data is a typical problem of spontaneous reporting, making it susceptible to reporting bias (Palleria et al., 2013). Indeed, we could not discern vestibular vertigo from the others, and we could not adjust ototoxicity for the effect of renal impairment. Besides, the lack of information about the total number of drug-exposed patients is a well-known limit of the spontaneous pharmacovigilance data that makes it impossible to calculate event rates in the absence of denominators (Pal et al., 2013).

## Conclusions

The present study highlights the importance of spontaneous reporting databases as a valid tool to detect rare and previously undocumented drug-induced ototoxic ADRs.

Our data are consistent with results from clinical trials and post-marketing data for quinolones, macrolides, aminoglycosides, urologicals, antimalarials, and antidepressants, such as for deferasirox, timolol used in combination for antiglaucoma therapy, somatropin agonists, antimigraine preparations, vancomycin, efavirenz, cisplatin, rituximab, interferon alfa-2b, thalidomide, tacrolimus, indomethacin, and etoricoxib. On the other hand, we observed disproportionate reporting about an unknown ototoxicity for propafenone, antituberculars, hormone antagonists, teriparatide, tramadol, and pomalidomide. Hypoacusis after the use of vinorelbine, methotrexate, and pemetrexed is unexpected, such as tinnitus related with etoposide, nebivolol, betamethasone, abatacept, sofosbuvir/ledipasvir, and tapentadol, but these require further investigation to better define the risk, due to the few reports available and to the frequent absence of clinical details useful to underline reporting of ADR evaluation. Physicians should be aware of the clinical significance of ototoxicity and its consequence on patients’ quality of life, and they should be conscious about the importance of their reporting to health authorities.

## Data Availability Statement

The datasets generated for this study will not be made publicly available. Restrictions apply to the datasets. The datasets for this manuscript are not publicly available because the pharmacovigilance data in single, non-aggregated form are available only under a specific authorization released by the Italian Medicine Agency. Requests to access the datasets should be directed to the Italian Medicine Agency.

## Ethics Statement

The study was approved by the Ethical Committee of Messina.

## Author Contributions

All authors listed have sufficiently made contributions to the entire content of the manuscript and have given their consent for publication. Project coordination: ES. Acquisition of data: PC and ES. Analysis and interpretation of data: MB, GC, PC, EM, and VA. Clinical evaluation of data: FF, FG, and ES. Writing of the paper: MB, GC, PC, and EM. Critical revision: LS, FG, and ES. Final approval of the version to be published: MB, GC, PC, EM, LS, FF, FG, VA, and ES.

## Conflict of Interest

The authors declare that the research was conducted in the absence of any commercial or financial relationships that could be construed as a potential conflict of interest.
