# Supplementary material for: Ototoxic Adverse Drug Reactions: A Disproportionality Analysis Using the Italian Spontaneous Reporting Database
Source: Front Pharmacol. 2019 Oct 8;10:1161. doi: 10.3389/fphar.2019.01161 (PMC6791930; doi:10.3389/fphar.2019.01161)
Supplement: Supplementary file 1 [file Table_1.docx]

**Supplementary Table 1.** Preferred Term (PT) affected by ADRs and drug classes mostly involved.

| **Preferred Term (PT)^a^** | ***n* (%)^b^** | **Drug classes (ATC III)^c^** |
| --- | --- | --- |
| Tinnitus | 358 (54.9) | L01X-other antineoplastic agents (33); M01A -antiinflammatory and antirheumatic products non–steroids (23); B01A-antithrombotic agents (22) |
| Hypoacusis (incl. neurosensory hypoacusis) | 213 (32.7) | L01X-other antineoplastic agents (42); L04A-immunosuppressants (22); J01F-macrolides, lincosamides and streptogramins (16); L01C- plant alkaloids and other natural products (16) |
| Vertigo positional | 34 (5.2) | N03A-antiepileptics (7); N06A –antidepressants (5); J04A-drugs for treatment of tuberculosis (3); N02A-opioids (3); B01A-antithrombotic agents (3) |
| Auditory disorder | 23 (3.5) | N05A-antipsychotics (5); J01M-quinolone antibacterials (3); M01A-antiinflammatory and antirheumatic products non–steroids (3) |
| Deafness (incl. deafness bilateral, deafness neurosensory, deafness unilateral and sudden hearing loss) | 22 (3.4) | L01X-other antineoplastic agents (4); J01G-aminoglycoside antibacterials (3); J01M-quinolone antibacterials (2); J01X-other antibacterials (2); G04B-urologicals (2) |

*ADR* Adverse Drug Reaction, *ATC* Anatomical Therapeutic Chemical Classification System

*^a^Only Preferred Term (PT) accounting for twenty or more reports related to ototoxicity were considered*

*^b^The sum of the distribution of ototoxic ADR reports by PT (%) is higher than the total number of reports, since a single report could contain more than one ADRs related to the selected PTs*

*^c^Only top three drug classes (n>1) reported for each PT were considered. In some cases, more drug categories were simultaneously involved*
