# Supplementary material for: Ototoxic Adverse Drug Reactions: A Disproportionality Analysis Using the Italian Spontaneous Reporting Database
Source: Front Pharmacol. 2019 Oct 8;10:1161. doi: 10.3389/fphar.2019.01161 (PMC6791930; doi:10.3389/fphar.2019.01161)
Supplement: Supplementary file 2 [file Table_2.docx]

**Supplementary Table 2.** Crude and adjusted Reporting Odds Ratio (ROR) for the association of drug classes with ototoxicity.

| **Drug Classes (ATC III)** | **Ototoxic ADR**  **reports (*n*)^a^** | **Other ADR reports (*n*)** | **Crude ROR**  **(95% CI)** | **Adjusted ROR ^b^**  **(95% CI)** |
| --- | --- | --- | --- | --- |
| L01X - other antineoplastic agents | 72 | 35.482 | 1.01 (0.79 - 1.30) | 1.03 (0.80 - 1.33) |
| L04A - immunosuppressants | 41 | 18.326 | 1.12 (0.82 - 1.54) | 1.21 (0.88 - 1.67) |
| J01M - quinolone antibacterials | 32 | 9.751 | 1.67 (1.17 - 2.38) | 1.61 (1.12 - 2.33) |
| N06A - antidepressants | 30 | 6.605 | 2.33 (1.61 - 3.36) | 2.35 (1.61 - 3.45) |
| B01A - antithrombotic agents | 29 | 39.387 | 0.34 (0.23 - 0.49) | 0.33 (0.22 - 0.48) |
| M01A - antiinflammatory and antirheumatic products non-steroids | 29 | 20.968 | 0.68 (0.47 - 0.98) | 0.66 (0.45 - 0.97) |
| L01C - plant alkaloids and other natural products | 27 | 9.367 | 1.46 (0.99 - 2.14) | 1.56 (1.06 - 2.30) |
| J01F - macrolides, lincosamides and streptogramins | 24 | 5.003 | 2.45 (1.63 - 3.68) | 2.54 (1.69 - 3.82) |
| J05A - direct acting antivirals | 23 | 7.755 | 1.50 (0.99 - 2.27) | 1.50 (0.98 - 2.30) |
| N03A - antiepileptics | 23 | 6.983 | 1.67 (1.01 - 2.53) | 1.39 (0.87 - 2.23) |
| J01G - aminoglycoside antibacterials | 20 | 371 | 27.72 (17.56 - 43.76) | 29.16 (18.44 - 46.10) |
| N02A - opioids | 19 | 6.948 | 1.38 (0.87 - 2.17) | 1.38 (0.86 - 2.21) |
| C07A - beta blocking agents | 18 | 3.277 | 2.79 (1.74 - 4.46) | 2.84 (1.75 - 4.61) |
| C10A - lipid modifying agents, plain | 17 | 7.564 | 1.13 (0.69 - 1.82) | 1.20 (0.74 - 1.94) |
| L03A - immunostimulants | 17 | 6.681 | 1.28 (0.79 - 2.07) | 1.35 (0.81 - 2.26) |
| C09A - ACE inhibitors, plain | 16 | 5.069 | 1.59 (0.97 - 2.61) | 1.67 (1.01 - 2.74) |
| L01B - antimetabolites | 16 | 11.013 | 0.72 (0.44 - 1.18) | 0.76 (0.46 - 1.24) |
| N05A - antipsychotics | 13 | 8.788 | 0.73 (0.42 - 1.27) | 0.77 (0.44 - 1.33) |
| J01C - beta–lactam antibacterials, penicillins | 12 | 21.427 | 0.27 (0.15 - 0.47) | 0.27 (0.15 - 0.47) |
| A02B - drugs for peptic ulcer and gastroesophageal reflux disease | 12 | 4.405 | 1.37 (0.77 - 2.42) | 1.20 (0.64 - 2.25) |
| C09C - agents acting on the renin-angiotensin system | 11 | 2.176 | 2.55 (1.40 - 4.63) | 2.70 (1.49 - 4.92) |
| C08C - selective Ca^2+^channel blockers with mainly vascular effect | 11 | 2.733 | 2.03 (1.12 - 3.68) | 2.14 (1.18 - 3.90) |
| L02B - hormone antagonists and related agents | 11 | 2.651 | 2.09 (1.15 - 3.80) | 2.03 (1.08 - 3.79) |
| V03A - all other therapeutic products | 11 | 1.214 | 4.58 (2.52 - 8.34) | 4.56 (2.43 - 8.54) |
| G04B - urologicals | 10 | 942 | 5.36 (2.86 - 10.05) | 4.78 (2.37 - 9.63) |
| J01X - other antibacterials | 9 | 2.570 | 1.76 (0.91 - 3.40) | 1.86 (0.96 - 3.60) |
| S01E - antiglaucoma preparations and miotics | 8 | 778 | 5.18 (2.57 - 10.44) | 4.94 (2.34 - 10.44) |
| V08A -  X–ray contrast media, iodinated | 7 | 9.848 | 0.35 (0.17 - 0.73) | 0.34 (0.16 - 0.73) |
| H05A - parathyroid hormones and analogues | 7 | 1.905 | 1.84 (0.87 - 3.89) | 2.24 (1.06 - 4.74) |
| H02A - corticosteroids for systemic use, plain | 6 | 3.297 | 0.91 (0.41 - 2.03) | 0.97 (0.43 - 2.18) |
| C03C - high–ceiling diuretics | 5 | 1.884 | 1.33 (0.55 - 3.20) | 1.48 (0.61 - 3.58) |
| M05B - drugs affecting bone structure and mineralization | 5 | 3.897 | 0.64 (0.26 - 1.54) | 0.70 (0.29 - 1.68) |
| R06A - antihistamines for systemic use | 5 | 1.328 | 1.89 (0.78 - 4.55) | 1.92 (0.80 - 4.64) |
| N02B - other analgesics and antipyretics | 5 | 7.802 | 0.32 (0. 13 - 0.76) | 0.32 (0.13 - 0.77) |
| C09D - angiotensin II antagonists, combinations | 5 | 1.866 | 1.34 (0.56 - 3.23) | 1.41 (0.58 - 3.40) |
| C02C - antiadrenergic agents, peripherally acting | 5 | 715 | 3.51 (1.45 - 8.48) | 3.69 (1.53 - 8.93) |
| J04A - drugs for treatment of tuberculosis | 5 | 999 | 2.51 (1.04 - 6.06) | 2.62 (1.09 - 6.34) |
| P01B - antimalarials | 4 | 675 | 2.97 (1.11 - 7.96) | 3.15 (1.17 - 8.43) |
| A10B - blood glucose lowering drugs, excl. insulins | 4 | 6.850 | 0.29 (0.11 - 0.77) | 0.30 (0.11 - 0.80) |
| G04C - drugs used in benign prostatic hypertrophy | 4 | 1.527 | 1.31 (0.49 - 3.50) | 1.29 (0.48 - 3.45) |
| C01B - antiarrhythmics, class I and III | 4 | 2.090 | 0.96 (0.36 - 2.55) | 1.01 (0.38 - 2.70) |
| N05C - hypnotics and sedatives | 4 | 2.384 | 0.84 (0.31 - 2.24) | 0.88 (0.33 - 2.36) |
| J02A - antimycotics for systemic use | 4 | 1.180 | 1.70 (0.63 - 4.54) | 1.33 (0.43 - 4.13) |
| N05B - anxiolytics | 4 | 4.290 | 0.46 (0.17 - 1.24) | 0.49 (0.18 - 1.31) |
| L01A - alkylating agents | 4 | 2.986 | 0.67 (0.25 - 1.78) | 0.73 (0.27 - 1.95) |
| N02C - antimigraine preparations | 4 | 637 | 3.15 (1.17 - 8.44) | 3.32 (1.24 - 8.92) |
| C09B - ACE inhibitors, combinations | 4 | 1.783 | 1.12 (0.42 - 3.00) | 1.16 (0.43 - 3.10) |
| H03A - thyroid preparations | 3 | 599 | 2.51 (0.80 - 7.81) | 2.02 (0.50 - 8.14) |
| G03A - hormonal contraceptives for systemic use | 3 | 2.035 | 0.73 (0.24 - 2.29) | 0.95 (0.30 - 2.97) |
| S02D - other otologicals | 3 | 20 | 75.19 (22.29 - 253.64) | 53.87 (12.51 –231.90) |
| A16A - other alimentary tract and metabolism products | 3 | 697 | 2.15 (0.69 - 6.71) | 2.34 (0.75 - 7.30) |
| H01A - anterior pituitary lobe hormones and analogues | 3 | 520 | 2.89 (0.93 - 9.01) | 3.69 (1.18 - 11.52) |
| C03E - diuretics and potassium-sparing agents in combination | 3 | 682 | 2.20 (0.71 - 6.86) | 2.40 (0.77 - 7.48) |
| J01D - other beta–lactam antibacterials | 3 | 8.065 | 0.18 (0.06 - 0.57) | 0.19 (0.06 - 0.58) |
| M04A - antigout preparations | 3 | 2.115 | 0.71 (0.23 - 2.20) | 0.50 (0.13 - 2.01) |
| L01D - cytotoxic antibiotics and related substances | 3 | 2.809 | 0.53 (0.17 - 1.65) | 0.57 (0.18 - 1.79) |

*ADR* Adverse Drug Reaction, *ATC* Anatomical Therapeutic Chemical Classification System, *ROR* Reporting Odds Ratio, *CI* Confidence Interval

*^a^Only ototoxic ADR reports for three or more were considered*

*^b^Adjusted for age, sex and number of drugs*
