# Supplementary material for: Ototoxic Adverse Drug Reactions: A Disproportionality Analysis Using the Italian Spontaneous Reporting Database
Source: Front Pharmacol. 2019 Oct 8;10:1161. doi: 10.3389/fphar.2019.01161 (PMC6791930; doi:10.3389/fphar.2019.01161)
Supplement: Supplementary file 3 [file Table_3.docx]

**Supplementary Table 3.** Crude and adjusted Reporting Odds Ratio (ROR) for the association of active substances with ototoxicity.

| **Drug Classes (ATC III)** | **Active Substances** | **Ototoxic ADR reports (*n*)^a^** | **Other ADR reports (*n*)** | **Unadjusted ROR**  **(95% CI)** | **Adjusted ROR^b^**  **(95% CI)** |
| --- | --- | --- | --- | --- | --- |
| A02B - drugs for peptic ulcer and gastro-oesophageal reflux disease | omeprazole | 3 | 685 | 2.19 (0.70 - 6.83) | 2.30 (0.74 - 7.17) |
|  | lansoprazole | 3 | 1.299 | 1.15 (0.37 - 3.59) | 1.21 (0.39 - 3.76) |
| B01A - antithrombotic agents | warfarin | 5 | 13.630 | 0.18 (0.07 - 0.43) | 0.18 (0.07 - 0.43) |
|  | acetylsalicylic acid | 9 | 9.179 | 0.48 (0.25 - 0.93) | 0.48 (0.25 - 0.93) |
|  | clopidogrel | 9 | 4.199 | 1.07 (0.55 - 2.07) | 1.15 (0.60 - 2.23) |
| C01B - antiarrhythmics, class I and III | propafenone | 3 | 284 | 5.29 (1.69 - 16.55) | 5.56 (1.78 - 17.40) |
| C02C - antiadrenergic agents, peripherally acting | doxazosin | 5 | 708 | 3.54 (1.47 - 8.57) | 3.73 (1.54 - 9.03) |
| C03C - high-ceiling diuretics | furosemide | 3 | 1.746 | 0.86 (0.28 - 2.67) | 0.94 (0.30 - 2.94) |
| C03E - diuretics and potassium-sparing agents in combination | hydrochlorothiazide and potassium-sparing agents | 3 | 433 | 3.47 (1.11 - 10.82) | 3.72 (1.19 - 11.62) |
| C07A - beta blocking agents | metoprolol | 3 | 322 | 4.67 (1.49 - 14.58) | 4.97 (1.59 - 15.54) |
|  | bisoprolol | 6 | 981 | 3.07 (1.37 - 6.88) | 2.84 (1.17 - 6.87) |
|  | nebivolol | 5 | 595 | 4.22 (1.74 - 10.21) | 4.40 (1.82 - 10.66) |
| C08C - selective calcium channel blockers with mainly vascular effects | amlodipine | 3 | 1.437 | 1.04 (0.34 - 3.24) | 1.10 (0.35 - 3.41) |
| C09A - ACE inhibitors, plain | ramipril | 13 | 2.778 | 2.36 (1.36 - 4.10) | 2. 50 (1.44 - 4.33) |
| C09C - agents acting on the renin-angiotensin system | irbesartan | 7 | 362 | 9.74 (4.59 - 20.66) | 10.44 (4.91 - 22.17) |
| C09D - angiotensin II antagonists, combinations | losartan and diuretics | 3 | 231 | 6.51 (2.08 - 20.37) | 6.83 (2.18 - 21.40) |
| C10A - lipid modifying agents, plain | atorvastatin | 8 | 2.715 | 1.48 (0.73 - 2.97) | 1.58 (0.79 - 3.19) |
| G04B - urologicals | sildenafil | 3 | 172 | 8.74 (2.78 - 27.43) | 5.96 (1.47 - 24.14) |
|  | tadalafil | 5 | 309 | 8.13 (3.35 - 19.73) | 9.36 (3.85 - 22.76) |
| H02A - corticosteroids for systemic use, plain | betamethasone | 3 | 714 | 2.10 (0.67 - 6.55) | 2.20 (0.71 - 6.85) |
| H05A - parathyroid hormones and analogues | teriparatide | 7 | 1.899 | 1.85 (0.88 - 3.90) | 2.25 (1.06 - 4.76) |
| J01C - beta-lactam antibacterials. penicillins | amoxicillin | 3 | 6.425 | 0.23 (0.07 - 0.71) | 0.23 (0.07 - 0.71) |
|  | amoxicillin and beta-lactamase inhibitor | 6 | 13.236 | 0.22 (0.10 - 0.49) | 0.22 (0.10 - 0.49) |
| J01F - macrolides. lincosamides and streptogramins | clarithromycin | 13 | 2.874 | 2.28 (1.32 - 3.96) | 2.34 (1.35 - 4.06) |
|  | azithromycin | 11 | 1.297 | 4.29 (2.36 - 7.80) | 4.47 (2.45 - 8.13) |
| J01G - aminoglycoside antibacterials | amikacin | 16 | 119 | 68.75 (40.56 - 116.53) | 75.05 (44.07 - 127.79) |
| J01M - quinolone antibacterials | ciprofloxacin | 10 | 3.020 | 1.66 (0.89 - 3.11) | 1.37 (0.68 - 2.75) |
|  | levofloxacin | 12 | 5.184 | 1.16 (0.65 - 2.05) | 1.20 (0.68 - 2.12) |
|  | moxifloxacin | 4 | 816 | 2.46 (0.92 - 6.58) | 2.53 (0.94 - 6.77) |
|  | prulifloxacin | 4 | 237 | 8.47 (3.14 - 22.82) | 8.64 (3.21 - 23.30) |
| J01X - other antibacterials | vancomycin | 4 | 671 | 2.99 (1.11 - 8.01) | 3.13 (1.17 - 8.40) |
| J04A - drugs for treatment of tuberculosis | ethambutol | 4 | 252 | 7.96 (2.96 - 21.45) | 8.60 (3.18 - 23.22) |
|  | rifampicin and isoniazid | 3 | 142 | 10.59 (3.37 - 33.30) | 10.97 (3.48 - 34.52) |
| J05A - direct acting antivirals | efavirenz | 3 | 171 | 8.79 (2.80 - 27.60) | 8.86 (2.82 - 27.85) |
|  | ribavirin | 6 | 3.379 | 0.89 (0.40 - 1.98) | 0.92 (0.41 - 2.05) |
|  | sofosbuvir and ledipasvir | 3 | 512 | 2.93 (0.94 - 9.15) | 3.19 (1.02 - 9.95) |
| L01B - antimetabolites | methotrexate | 3 | 1.507 | 0.99 (0.32 - 3.09) | 1.09 (0.35 - 3.41) |
|  | pemetrexed | 5 | 1.101 | 2.28 (0.94 - 5.50) | 2.32 (0.96 - 5.60) |
|  | fluorouracil | 5 | 3.362 | 0.68 (0.28 - 1.64) | 0.73 (0.30 - 1.75) |
| L01C - plant alkaloids and other natural products | vincristine | 4 | 713 | 2.81 (1.05 - 7.53) | 3.08 (1.15 - 8.29) |
|  | vinorelbine | 5 | 390 | 6.44 (2.66 - 15.61) | 6.75 (2.78 - 16.36) |
|  | etoposide | 4 | 569 | 3.52 (1.31 - 9.45) | 3.71 (1.38 - 9.96) |
|  | paclitaxel | 10 | 4.521 | 1.11 (0.59 - 2.07) | 1.20 (0.64 - 2.25) |
|  | docetaxel | 4 | 2.488 | 0.80 (0.30 - 2.13) | 0.84 (0.31 - 2.24) |
| L01X - other antineoplastic agents | cisplatin | 34 | 1.731 | 10.29 (7.26 - 14.57) | 10.16 (7.09 - 14.56) |
|  | rituximab | 5 | 2.036 | 1.23 (0.51 - 2.96) | 1.30 (0.54 - 3.14) |
|  | carboplatin | 5 | 2.488 | 1.00 (0.42 - 2.42) | 1.08 (0.45 - 2.60) |
|  | oxaliplatin | 6 | 5.281 | 0.56 (0.25 - 1.26) | 0.58 (0.26 - 1.30) |
|  | bortezomib | 3 | 910 | 1.65 (0.53 - 5.13) | 1.71 (0.55 - 5.34) |
| L02B - hormone antagonists and related agents | tamoxifen | 3 | 265 | 5.67 (1.81 - 17.74) | 6.33 (2.02 - 19.85) |
|  | anastrozole | 4 | 476 | 4.21 (1.57 - 11.31) | 3.45 (1.10 - 10.79) |
| L03A - immunostimulants | interferon alfa-2b | 9 | 724 | 6.28 (3.24 - 12.17) | 6.43 (3.31 - 12.47) |
|  | glatiramer acetate | 5 | 1.258 | 1.99 (0.82 - 4.81) | 1.46 (0.47 - 4.56) |
| L04A - immunosuppressants | abatacept | 4 | 823 | 2.43 (0.91 - 6.52) | 3.67 (1.37 - 9.87) |
|  | infliximab | 4 | 2.239 | 0.89 (0.33 - 2.38) | 0.92 (0.35 - 2.48) |
|  | adalimumab | 4 | 1.669 | 1.20 (0.45 - 3.20) | 1.36 (0.51 - 3.64) |
|  | tacrolimus | 3 | 152 | 9.89 (3.15 - 31.09) | 10.55 (3.35 - 33.22) |
|  | thalidomide | 7 | 535 | 6.59 (3.11 - 13.94) | 7.44 (3.51 - 15.76) |
|  | lenalidomide | 3 | 3.444 | 0.43 (0.14 - 1.34) | 0.45 (0.15 - 1.40) |
|  | pomalidomide | 3 | 173 | 8.69 (2.77 - 27.27) | 10.64 (3.38 - 33.48) |
| M01A - antiinflammatory and antirheumatic products, non-steroids | indometacin | 4 | 449 | 4.47 (1.66 - 11.99) | 4.71 (1.76 - 12.65) |
|  | diclofenac | 4 | 3.100 | 0.64 (0.24 - 1.72) | 0.49 (0.16 - 1.54) |
|  | ketoprofen | 3 | 5.077 | 0.29 (0.09 - 0.91) | 0.29 (0.09 - 0.90) |
|  | etoricoxib | 6 | 1.130 | 2.67 (1.19 - 5.97) | 2.77 (1.24 - 6.21) |
| M04A - antigout preparations | allopurinol | 3 | 1.826 | 0.82 (0.26 - 2.55) | 0.58 (0.15 - 2.34) |
| N02A - opioids | tramadol | 8 | 2.031 | 1.98 (0.98 - 3.98) | 2.07 (1.03 - 4.17) |
|  | tapentadol | 3 | 461 | 3.26 (1.04 - 10.16) | 2.45 (0.61 - 9.83) |
| N02B - other analgesics and antipyretics | acetylsalicylic acid | 5 | 1.523 | 1.64 (0.68 - 3.97) | 1.64 (0.68 - 3.96) |
| N03A - antiepileptics | carbamazepine | 3 | 1.075 | 1.39 (0.45 - 4.34) | 0.98 (0.24 - 3.92) |
|  | oxcarbazepine | 3 | 388 | 3.87 (1.24 - 12.09) | 2.75 (0.68 - 11.06) |
|  | valproic acid | 5 | 1.205 | 2.08 (0.86 - 5.02) | 1.75 (0.65 - 4.69) |
|  | levetiracetam | 3 | 538 | 2.79 (0.90 - 8.70) | 3.03 (0.97 - 9.46) |
|  | pregabalin | 3 | 1.037 | 1.45 (0.46 - 4.50) | 1.05 (0.26 - 4.22) |
| N05A - antipsychotics | quetiapine | 3 | 1.766 | 0.85 (0.27 - 2.64) | 0.91 (0.29 - 2.82) |
| N06A - antidepressants | paroxetine | 6 | 996 | 3.02 (1.35 - 6.77) | 2.67 (1.10 - 6.45) |
|  | duloxetine | 5 | 722 | 3.47 (1.44 - 8.40) | 3.11 (1.16 - 8.33) |
|  | escitalopram | 4 | 699 | 2.87 (1.07 - 7.68) | 3.15 (1.17 - 8.44) |
|  | sertraline | 5 | 893 | 2.81 (1.16 - 6.79) | 2.99 (1.24 - 7.24) |
|  | vortioxetine | 3 | 141 | 10.66 (3.39 - 33.54) | 13.18 (4.18 - 41.58) |
| S01E - antiglaucoma preparations and miotics | timolol, combinations | 5 | 216 | 11.63 (4.78 - 28.32) | 12.70 (5.21 - 30.95) |
| V03A - all other therapeutic products | deferasirox | 10 | 328 | 15.43 (8.19 - 29.09) | 14.66 (7.52 - 28.58) |
| V08A - X-ray contrast media, iodinated | iomeprol | 3 | 3.714 | 0.40 (0.13 - 1.25) | 0.40 (0.13 - 1.23) |

*ADR* Adverse Drug Reaction, *ATC* Anatomical Therapeutic Chemical Classification System, *ROR* Reporting Odds Ratio, *CI* Confidence Interval

*^a^Only ototoxic ADR reports for three or more were considered*

*^b^Adjusted for age, sex and number of drugs*
