# Supplementary material for: Ototoxic Adverse Drug Reactions: A Disproportionality Analysis Using the Italian Spontaneous Reporting Database
Source: Front Pharmacol. 2019 Oct 8;10:1161. doi: 10.3389/fphar.2019.01161 (PMC6791930; doi:10.3389/fphar.2019.01161)
Supplement: Supplementary file 4 [file Table_4.docx]

**Supplementary Table 4.** Crude and adjusted Reporting Odds Ratio (ROR) for the association of active substances with tinnitus.

| **Drug Classes (ATC III)** | **Active Substances** | **Tinnitus reports (*n*)^a^** | **Other ADR reports (*n*)** | **Unadjusted ROR**  **(95% CI)** | **Adjusted ROR^b^**  **(95% CI)** |
| --- | --- | --- | --- | --- | --- |
| B01A - antithrombotic agents | acetylsalicylic acid | 8 | 9.180 | 0.79 (0.39 – 1.59) | 0.80 (0.39 – 1.62) |
|  | clopidogrel | 7 | 4.201 | 1.53 (0.72 – 3.23) | 1.67 (0.79 – 3.53) |
| C02C - antiadrenergic agents, peripherally acting | doxazosin | 4 | 709 | 5.18 (1.93 – 13.91) | 5.55 (2.06 – 14.93) |
| C07A - beta blocking agents | bisoprolol | 5 | 982 | 4.68 (1.93 – 11.35) | 4.28 (1.59 – 11.53) |
|  | nebivolol | 5 | 595 | 7.74 (3.19 – 18.77) | 8.06 (3.32 – 19.56) |
| C08C - selective calcium channel blockers with mainly vascular effects | amlodipine | 3 | 1.437 | 1.91 (0.61 – 5.95) | 2.06 (0.65 – 6.35) |
| C09A - ACE inhibitors, plain | ramipril | 11 | 2.780 | 3.68 (2.02 – 6.72) | 3.96 (2.17 – 7.23) |
| C09C - agents acting on the renin-angiotensin system | irbesartan | 7 | 362 | 17.92 (8.42 - 38.13) | 19.60 (9.19 – 41.80) |
| C10A - lipid modifying agents, plain | atorvastatin | 6 | 2.717 | 2.03 (0.90 – 4.54) | 2.20 (0.98 – 4.94) |
| H02A - corticosteroids for systemic use, plain | betamethasone | 3 | 714 | 3.85 (1.23 – 12.01) | 4.01 (1.28 – 12.52) |
| J01C - beta-lactam antibacterials. penicillins | amoxicillin and beta-lactamase inhibitor | 5 | 13.237 | 0.33 (0.14 - 0.81) | 0.33 (0.14 - 0.80) |
| J01F - macrolides. lincosamides and streptogramins | clarithromycin | 5 | 2.882 | 1.59 (0.66 - 3.84) | 1.61 (0.67 - 3.91) |
| J01G - aminoglycoside antibacterials | amikacin | 5 | 130 | 35.46 (14.43 – 87.17) | 39.52 (16.02 – 97.52) |
| J01M - quinolone antibacterials | ciprofloxacin | 6 | 3.024 | 1.82 (0.81 – 4.08) | 1.24 (0.46 – 3.31) |
|  | levofloxacin | 5 | 5.191 | 0.87 (0.36 - 2.12) | 0.90 (0.37 - 2.17) |
|  | moxifloxacin | 4 | 816 | 4.50 (1.68 – 12.09) | 4.56 (1.71 – 12.34) |
| J04A - drugs for treatment of tuberculosis | ethambutol | 3 | 253 | 10.87 (3.47 – 34.09) | 12.25 (3.89 – 38.57) |
| J05A - direct acting antivirals | efavirenz | 3 | 171 | 16.08 (5.11 – 50.60) | 16.82 (5.34 – 52.96) |
|  | sofosbuvir and ledipasvir | 3 | 512 | 5.37 (1.72 – 16.77) | 5.95 (1.90 – 18.61) |
| L01C - plant alkaloids and other natural products | etoposide | 4 | 569 | 6.46 (2.40 – 17.35) | 7.09 (2.63 – 19.12) |
|  | paclitaxel | 3 | 4.528 | 0.60 (0.19 – 1.87) | 0.65 (0.21 - 2.02) |
|  | docetaxel | 3 | 2.489 | 1.09 (0.35 – 3.40) | 1.15 (0.37 – 3.58) |
| L01X - other antineoplastic agents | cisplatin | 13 | 1.752 | 6.97 (4.00 – 12.14) | 6.18 (3.38 – 11.30) |
|  | oxaliplatin | 4 | 5.283 | 0.69 (0.26 - 1.84) | 0.71 (0.26 - 1.90) |
| L03A - immunostimulants | glatiramer acetate | 5 | 1.258 | 3.65 (1.51 – 8.84) | 2.55 (0.82 – 7.96) |
| L04A - immunosuppressants | abatacept | 4 | 823 | 4.46 (1.66 – 11.98) | 6.51 (2.42 – 17.53) |
|  | adalimumab | 3 | 1.670 | 1.64 (0.53 – 5.11) | 1.85 (0.59 – 5.77) |
| M01A - antiinflammatory and antirheumatic products, non-steroids | indometacin | 3 | 450 | 6.11 (1.95 – 19.10) | 6.30 (2.02 – 19.72) |
|  | ketoprofen | 3 | 5.077 | 0.53 (0.17 – 1.66) | 0.53 (0.17 – 1.64) |
|  | etoricoxib | 6 | 1.130 | 4.90 (2.18 – 10.99) | 5.00 (2.23 – 11.23) |
| N02A - opioids | tramadol | 4 | 2.035 | 1.80 (0.67 – 4.82) | 1.88 (0.70 – 5.05) |
|  | tapentadol | 3 | 461 | 5.96 (1.91 – 18.64) | 4.37 (1.09 – 17.62) |
| N02B - other analgesics and antipyretics | acetylsalicylic acid | 3 | 1.525 | 1.80 (0.58 – 5.60) | 1.81 (0.58 – 5.66) |
| N06A - antidepressants | paroxetine | 6 | 996 | 5.56 (2.47 – 12.48) | 4.79 (1.98 – 11.61) |
|  | sertraline | 4 | 894 | 4.10 (1.53 – 11.02) | 4.34 (1.61 – 11.65) |
| S01E - antiglaucoma preparations and miotics | timolol, combinations | 5 | 216 | 21.34 (8.74 – 52.11) | 23.29 (9.53 – 56.95) |

*ADR* Adverse Drug Reaction, *ATC* Anatomical Therapeutic Chemical Classification System, *ROR* Reporting Odds Ratio, *CI* Confidence Interval

*^a^Only tinnitus related ADR reports for three or more were considered*

*^b^Adjusted for age, sex and number of drugs*
