# Supplementary material for: Ototoxic Adverse Drug Reactions: A Disproportionality Analysis Using the Italian Spontaneous Reporting Database
Source: Front Pharmacol. 2019 Oct 8;10:1161. doi: 10.3389/fphar.2019.01161 (PMC6791930; doi:10.3389/fphar.2019.01161)
Supplement: Supplementary file 5 [file Table_5.docx]

**Supplementary Table 5.** Crude and adjusted Reporting Odds Ratio (ROR) for the association of active substances with hypoacusis.

| **Drug Classes (ATC III)** | **Active Substances** | **Hypoacusis reports (*n*)^a^** | **Other ADR reports (*n*)** | **Unadjusted ROR**  **(95% CI)** | **Adjusted ROR^b^**  **(95% CI)** |
| --- | --- | --- | --- | --- | --- |
| C03C - high-ceiling diuretics | furosemide | 3 | 1.746 | 2.65 (0.85 – 8.30) | 3.13 (0.99 – 9.88) |
| J01F - macrolides. lincosamides and streptogramins | clarithromycin | 7 | 2880 | 3.81 (1.79 – 8.10) | 3.95 (1.86 – 8.40) |
|  | azithromycin | 8 | 1.300 | 9.74 (4.80 – 19.78) | 10.23 (5.03 – 20.79) |
| J01G - aminoglycoside antibacterials | amikacin | 10 | 125 | 128.33 (66.42 – 247.96) | 128.65 (66.37 – 249.37) |
| J01M - quinolone antibacterials | levofloxacin | 3 | 5.193 | 0.88 (0.28 – 2.76) | 0.93 (0.30 - 2.90) |
| J01X - other antibacterials | vancomycin | 3 | 672 | 6.91 (2.21 – 21.66) | 6.72 (2.14 – 21.11) |
| J05A - direct acting antivirals | ribavirin | 5 | 3.330 | 2.29 (0.94 – 5.57) | 2.32 (0.96 – 5.65) |
| L01B - antimetabolites | methotrexate | 3 | 1.507 | 3.07 (0.98 – 9.62) | 3.13 (1.00 – 9.81) |
|  | pemetrexed | 3 | 1.103 | 4.21 (1.34 – 13.16) | 4.38 (1.40 – 13.76) |
|  | fluorouracil | 5 | 3.362 | 2.11 (0.87 – 5.14) | 2.21 (0.91 – 5.40) |
| L01C - plant alkaloids and other natural products | vincristine | 3 | 714 | 6.50 (2.08 – 20.38) | 5.93 (1.88 – 18.70) |
|  | vinorelbine | 5 | 390 | 20.06 (8.22 – 48.96) | 21.60 (8.83 – 52.82) |
|  | paclitaxel | 6 | 4.525 | 2.06 (0.91 – 4.64) | 2.34 (1.03 – 5.30) |
| L01X - other antineoplastic agents | cisplatin | 19 | 1.746 | 18.18 (11.32 – 29.18) | 19.29 (11.99 – 31.03) |
|  | rituximab | 4 | 2.037 | 3.04 (1.13 – 8.19) | 3.20 (1.19 – 8.63) |
|  | carboplatin | 3 | 2.490 | 1.86 (0.59 – 5.80) | 2.08 (0.66 – 6.53) |
|  | oxaliplatin | 3 | 5.284 | 0.87 (0.28 – 2.71) | 0.91 (0.29 – 2.85) |
| L03A - immunostimulants | interferon alfa-2b | 8 | 725 | 17.50 (8.60 – 35.59) | 17.44 (8.56 – 35.53) |
| L04A - immunosuppressants | thalidomide | 5 | 537 | 14.56 (5.97 – 35.49) | 16.92 (6.92 – 41.38) |
|  | lenalidomide | 3 | 3.444 | 1.34 (0.43 – 4.18) | 1.51 (0.48 – 4.75) |
| V03A - all other therapeutic products | deferasirox | 9 | 329 | 43.64 (22.19 – 85.82) | 41.06 (20.07 – 84.01) |

*ADR* Adverse Drug Reaction, *ATC* Anatomical Therapeutic Chemical Classification System, *ROR* Reporting Odds Ratio, *CI* Confidence Interval

*^a^Only ototoxic ADR reports for three or more were considered*

*^b^Adjusted for age, sex and number of drugs*
